# Handling climate change education at universities: an overview

**DOI:** 10.1186/s12302-021-00552-5

**Published:** 2021-09-25

**Authors:** Walter Leal Filho, Mihaela Sima, Ayyoob Sharifi, Johannes M. Luetz, Amanda Lange Salvia, Mark Mifsud, Felicia Motunrayo Olooto, Ilija Djekic, Rosley Anholon, Izabela Rampasso, Felix Kwabena Donkor, Maria Alzira Pimenta Dinis, Maris Klavins, Göran Finnveden, Martin Munashe Chari, Petra Molthan-Hill, Alexandra Mifsud, Salil K. Sen, Erandathie Lokupitiya

**Affiliations:** 1 School of Science and the Environment, Manchester Metropolitan University, Chester Street, Manchester, M1 5GD UK; 2 Research and Transfer Centre “Sustainable Development and Climate Change Management”, Hamburg University of Applied Sciences, Hamburg, Germany; 3 Romanian Academy, Institute of Geography, 12 Dimitrie Racovita St, Sector 2, 023993 Bucharest, Romania; 4 Graduate School of Humanities and Social Sciences, and Network for Education and Research on Peace and Sustainability, Hiroshima University, Higashi-Hiroshima, 739-8530 Japan; 5 School of Social Sciences, University of New South Wales (UNSW), Sydney, Australia; 6 Christian Heritage College (CHC), Brisbane, Australia; 7 School of Law and Society, University of the Sunshine Coast (USC), Maroochydore, Australia; 8 Graduate Program in Civil and Environmental Engineering, University of Passo Fundo, Passo Fundo, Brazil; 9 University of Malta, Msida, Malta; 10 Department of Agricultural Economics and Extension Services, Faculty of Agriculture, Kwara State University, Malete, PMB 1530, Ilorin, Kwara State Nigeria; 11 Faculty of Agriculture, University of Belgrade, Belgrade, Republic of Serbia; 12 University of Campinas, Campinas, Brazil; 13 Universidad Católica del Norte, Departamento de Ingeniería Industrial, Angamos, 0610, Antofagasta, Chile; 14 PNPD/CAPES Program, Doctoral Program in Sustainable Management Systems, Federal Fluminense University, Brazil, Passo da Pátria Street, 156, Niterói, Brazil; 15 Department of Geography Education, University of Education Winneba, Winneba, Ghana; 16 UFP Energy, Environment and Health Research Unit (FP-ENAS), University Fernando Pessoa (UFP), Praça 9 de Abril 349, 4249-004 Porto, Portugal; 17 Department of Environmental Science, University of Latvia, Raina blvd 19, Riga, LV 1586 Latvia; 18 Department of Sustainable Development, Environmental Sciences and Engineering, KTH Royal Institute of Technology, Stockholm, Sweden; 19 Environmental Sustainability Assessment and Circularity, Luxembourg Institute of Science and Technology, Belvaux, Luxembourg; 20 Risk and Vulnerability Science Centre (RVSC), Faculty of Science and Agriculture, University of Fort Hare, 1 King William’s Town Road, Private Bag X1314, Alice, 5700 Eastern Cape South Africa; 21 Nottingham Business School, Nottingham Trent University, Nottingham, UK; 22 Nottingham Trent University, Nottingham, UK; 23 Management Development Institute of Singapore, 501 Stirling Rd, Singapore, 148951 Singapore; 24 Indian Institute of Management, Jingkieng, Nongthymmai, Shillong, Meghalaya 793014 India; 25 Department of Zoology and Environment Sciences, Faculty of Science, University of Colombo, Colombo 03, Sri Lanka

**Keywords:** Climate change education, Universities, Training needs, Bibliometric analysis, Online worldwide survey, Case studies, Climate change teaching and research

## Abstract

**Background:**

Climate change is a problem which is global in nature, and whose effects go across a wide range of disciplines. It is therefore important that this theme is taken into account as part of universities´ teaching and research programs.

**Methods:**

A three-tiered approach was used, consisting of a bibliometric analysis, an online survey and a set of case studies, which allow a profile to be built, as to how a sample of universities from 45 countries handle climate change as part of their teaching programs.

**Results:**

This paper reports on a study which aimed at identifying the extent to which matters related to climate change are addressed within the teaching and research practices at universities, with a focus on the training needs of teaching staff. It consists of a bibliometric analysis, combined with an online worldwide survey aimed at ascertaining the degree of involvement from universities in reducing their own carbon footprint, and the ways they offer training provisions on the topic. This is complemented by a set of 12 case studies from universities round the world, illustrating current trends on how universities handle climate change. Apart from reporting on the outcomes of the study, the paper highlights what some universities are doing to handle climate issues, and discusses the implications of the research.

**Conclusions:**

The paper lists some items via which universities may better educate and train their students on how to handle the many challenges posed by climate change.

**Supplementary Information:**

The online version contains supplementary material available at 10.1186/s12302-021-00552-5.

## Introduction

### Climate change and education

**Fig. 1:**
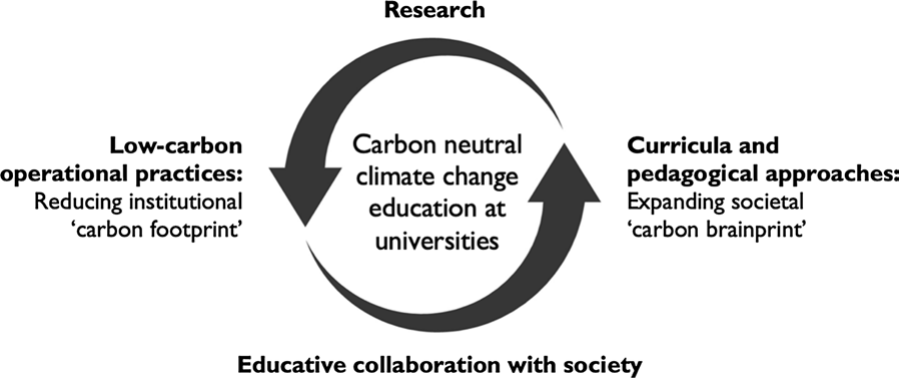
Schematic concept of holistic climate change education at universities comprising research, low-carbon operational practices, forms of educative collaboration with society, and curricula and pedagogy. Figure by authors, based on Chatterton et al. [11] and Baumber et al. [4]

Universities globally are increasingly recognizing their responsibility to prepare students and society to actively contribute to the mitigation of and adaptation to climate change. This role sees universities adopting and promoting carbon neutral goals and practices [13, 24, 57]. Situated within this broader context, contemporary higher education (HE) providers progressively pursue a dual strategy [4]. First, universities are aiming to become “carbon neutral” institutions by adopting low-carbon operational practices. Second, universities are developing curricula and pedagogical approaches to educate students (and by extension society) about the imperatives of carbon neutrality and climate change mitigation and adaptation. In the literature this dual education has been conceptualized as a critical twin strategy that sees universities concurrently reduce their own “carbon footprint” (by aiming for net-zero emissions of institution-linked greenhouse gasses) and expanding the societal “carbon brainprint” (by teaching knowledge and skills in the area of carbon neutral practices) [4, 11, 24] (Fig. 1). As such, HE providers have a vital role to play in educating future environmental auditors, community organizers, corporate managers, engineers, practitioners, technical professionals, policymakers and, most significantly, the community about actions that can be taken to mitigate and adapt to climate change, while concurrently propagating social and governance measures. Over time the cumulative build-up of societal awareness progressively permeates and influences the practices of the corporate sector, community stakeholders and local and national governments on how to better manage climate change mitigation and adaptation in their diverse spheres of influence, including through advocacy, daily behaviors and professional careers [13, 21, 57].

While universities are thus recognizing their responsibility to progressively prioritize carbon neutrality and develop climate change modules in educational content, efforts can sometimes be stymied by organizational inertia, operational complexity and a plethora of regulatory requirements that impinge on governance in the higher education sector [39, 57].

Climate change education (CCE) at universities may take the form of both formal, informal, and non-formal learning and teaching approaches, including nature-immersive field projects, international case studies and higher degree research (HDR), among others [39, 40, 69]. Literary analysis of university education on climate change and sustainability has reflected a gradual shift globally over the past decade away from a narrow preoccupation in curricula on environmental protection toward broader objectives and creative educational approaches. These initiatives include corporate social responsibility (CSR), multiculturality and ethics. They also manifest as renewable energy, recyclable resources, campus greening, embodied pedagogies, and nature-immersive outdoor education, among others [8, 35, 47, 70]. Molthan-Hill et al. [40] offer extensive guidance on how to integrate CCE across a wide range of subjects in university curricula, particularly ones with a less direct link to climate science; this guidance is offered from the point of view of content, pedagogy and contextual institutional and sector-wide constraints.

The way forward for universities is to dynamically reposition. This would measurably bridge the gap between generating direct action in the area of carbon neutral education and the creation and dissemination of critical knowledge about climate change that has the potential to proliferate across multiple other sectors [11, 13]. Corollary benefits for universities would be evident with significant influence on community leadership. Financial investments would apportion and support economic, environmental and societal value creation. Over recent years engagement in this space has marked an area of growing attention for higher education providers. Instances of universities taking the onus of investing in environmentally sustainable projects to attain sustainability objectives have escalated. Recent trends have additionally seen a growing number of universities globally take decisions to actively divest their endowments from fossil fuel holdings [4, 6]. Further, the pathway for climate driven education is integrative, strategic, and progressively embeds the Sustainable Development Goals (SDGs) [18, 60].

The Times Higher Education Impact Rankings is a tool used to describe the metrics used by universities in implementing individual UN SDGs [51]. Among them, SDG 13—climate action—is used. This measures whether universities perform research on climate change, how they use low-carbon energy, and if they have education programs aimed at the achievement of carbon neutrality. The latest list (for 2020) covers 376 universities from 70 countries. The top five universities are in New Zealand, Australia and the United States. These universities are the ones targeted in the current study.

## Climate change and universities

### The nexus of climate change education

Climate change and climate variability are principal issues confronting the global community [16]. The intricate nature of the global climate as an interconnected system, comprising earth and socio-ecological systems, necessitates critical enquiries coupled with reflexive and transformative educational methods [68]. This may help to address the need for more radical social learning-centered transformation in relation to sustainability concerns and the challenges posed to learning and pedagogy [18]. There is hence a need to shift from simple content-based, silo approaches of pedagogy to a more systemic and ‘deeper’ enquiry that draws together biophysical, socio-economic and socio-psychological understandings [23, 34]. In this light, concepts such as Education for Sustainable Development (ESD), as an integral component of quality education and critical tool for sustainable development has gained global currency [62]. ESD empowers people to change their thinking and approaches towards a sustainable future. This can be facilitated by enhancing opportunities for quality education on sustainable development [15]. This will promote social transformation through the redesigning of educational pedagogies and empowering people to build knowledge, skills, values and behaviors critical for sustainable development. This also underscores the need to integrate sustainable development themes, such as climate change within teaching and learning (UNESCO 2020). Efforts are needed from universities around the world to develop advanced curriculum, programs, capacity building and interdisciplinary collaboration in order to support a deeper learning on climate change [12, 48].

### The SDG final Decade of Action in delivering the global goals

United Nations (UN) studies show that although some progress has been made in a number of the SDGs, there is still substantial work to be done in achieving the SDGs, particularly in the area of redressing social inequity [14, 36]. The year 2020 marked the inception point of the Decade of Action to achieve the SDGS by 2030 [60]. The Decade is a rallying cry for the entire global community to catalyze actions in addressing urgent global challenges—from poverty and gender to climate change, and inequalities. The UN has encouraged all sectors of the society to rally their resources towards the Decade of Action in three core dimensions: global action to achieve effective leadership, improved resources and smarter solutions for realizing the SDGs [60]. In this regard, education and training will play a critical role. This is more so as climate-induced extreme events have been on the ascendancy across the globe, and argued to be reversing critical developmental gains [54]. It is important that all the advances and successes concerning the HE for sustainable development are safeguarded as much as possible [33]. The success of related measures is partly dependent on an informed and proactive citizenry cooperating with other core stakeholders. Higher education institutions (HEIs) are vital stakeholders who function along critical levers in the dynamics of sustainable development [53]. Furthermore, HEIs represent entrepreneurial spaces where social stakeholders collaborate in knowledge co-production to tackle pressing social issues. This article explores some of the ways higher education institutions can show leadership in climate education and research practices, as well as the inherent opportunities that can be upscaled in a post-COVID era for sustainability.

## Methodology

The paper aims to identify how matters related to climate change are tackled at universities both in teaching and research, with a focus on the training needs of teaching staff. It is developed in three directions: a bibliometric analysis, an online worldwide survey aimed at ascertaining the degree of involvement from universities to offer training provisions on the topic and a set of 12 case studies from universities round the world, illustrating current trends on how they handle climate change in teaching and research. These methods are mutually complementary for 3 main reasons: they provide an overview of the recent and current literature and their focus (bibliometric analysis); they enable the identification of trends among academic staff (the survey), and they cater for the provisions of real life examples illustrating how climate change is being implemented in university activities (case studies). Table 1 summarizes the purposes of each method and their contribution to this study.

**Table 1:** Methods used in the analysis

Method	Purpose	Details
Bibliometric analysis	Identifying trends in the study of CCE and its main focus in the literature	Database: Web of Science Software: VOSviewer Search string: ((“climat* change”) and (“education” or “training” or “curricula” or “curriculum”) AND (“universit*” OR “higher education institut*”)) Number of articles analyzed: 414
Survey	Providing a worldwide overview of the training needs on CCE at universities	Survey tool: SurveyMonkey Number of questions: 22 Number of respondents: 129 Number of countries: 45
Case studies	Illustrating current trends on how universities handle climate change in their activities	Number of cases: 12 Criteria: university ranking and geographic location Climate-related actions: i) accredited teaching programs of all levels of studies and research activities; ii) Various training events focused on staff and/or students; iii) public initiatives; and iv) other climate change-related activities

*Bibliometric methods* are increasingly used to understand structure and trends of scientific publications. The term co-occurrence analysis is a specific bibliometric analysis method that allows understanding overall structure and thematic focus of scientific fields. Several software tools have been developed over the past few years that can be used for detailed co-occurrence analysis of terms mentioned in publications that are indexed in scientific databases. In this study the VOSviewer has been used, a frequently utilized software tool for bibliometric analyses [67]. The input data for analysis were bibliometric information of peer-reviewed publications that are indexed in the Web of Science (WoS). WoS was selected for its broad coverage of high-quality scientific publications. It also provides detailed bibliographic information necessary for analysis using the VOSviewer. To collect relevant information from the WoS, a broad-based search string was developed that includes terms related to climate change, universities, and education/training: *TS* = ((“climat* change”) and (“education” or “training” or “curricula” or “curriculum”) AND (“universit*” OR “higher education institut*”)).

**Fig. 2:**
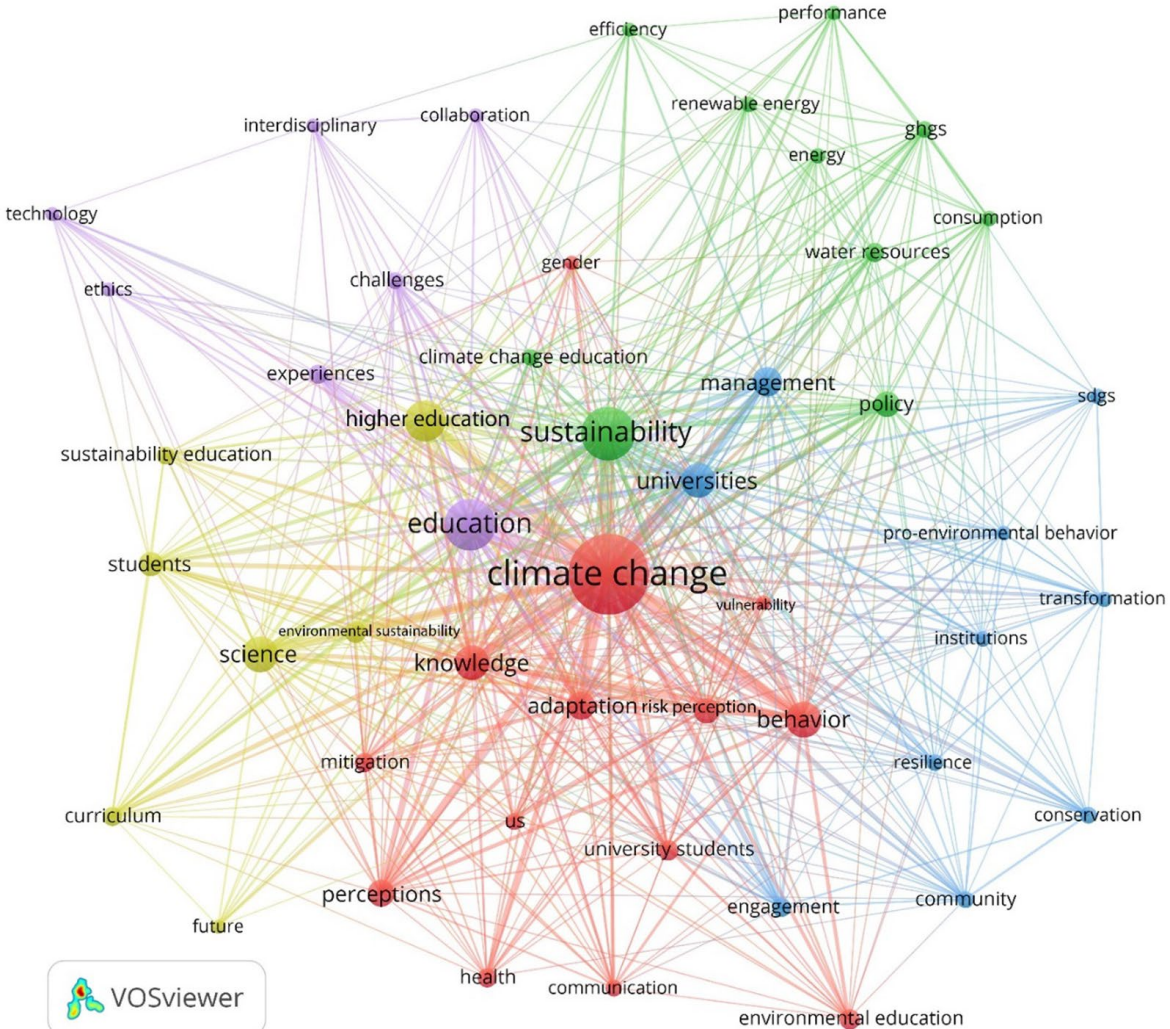
Output of the term co-occurrence analysis

The initial search was done on February 26, 2020 and returned 454 articles. After screening these articles to determine their relevance to the aims and objectives of the study, 414 articles remained in the database. In line with the study objectives, the criterion for inclusion in the analysis was addressing issues related to the integration of climate change in the teaching and research practices at universities. The bibliographic data of these articles were downloaded and used for term co-occurrence analysis using VOSviewer. Since multiple variants of a specific term may exist in the articles, a thesaurus file was created to merge synonyms. The output of the term co-occurrence analysis is a network of nodes and links (see Fig. 2), where the size of nodes and links indicates the frequency of occurrence and the strength of connections between nodes, respectively. Terms closely linked to each other form clusters that are shown in unique colors in Fig. 2. These clusters are specific thematic areas that will be discussed in the results section.

### The methodology of the survey

In order to establish the degree of involvement from universities in reducing their own carbon footprint and to fill in the research gap on how universities around the world handle climate change as part of their teaching programs, a questionnaire survey was undertaken. The questionnaire was part of an international study undertaken by the European School of Sustainability Science and Research (ESSSR) https://esssr.eu/ and the Inter University Sustainable Development Research Programme (IUSDRP) https://www.hawhamburg.de/ftz-nk/programme/iusdrp.html, aimed at identifying knowledge gaps on CCE at universities.

The first list of items was reviewed by the authors to minimize redundancies and similar items and to ensure that all important questions were added. The questionnaire survey was pre-tested by a panel of academics within sustainability areas at different universities.

The main target group of the questionnaire was the university staff involved in teaching and/or research at the university, who already integrated climate change-related aspects in a course at the university. The target individuals were identified through a number of mailing lists, including the European School of Sustainability Science and Research mailing list (https://esssr.eu/), JiscMail (https://www.jiscmail.ac.uk/) and the Inter-University Program for Sustainable Development Research (IUSDRP) list. The estimated response time was 10 min and no incentives were offered for questionnaire completion. At the end, 129 participants from 45 countries participated in the survey. The questionnaire was composed of 22 items, mainly closed-ended questions, divided into 3 major sections (Additional file 1: Appendix S1). The questions were formulated based on the authors’ knowledge and experience in terms of CCE, but also considering the results and training needs identified in other studies, targeting a single university or global studies (e.g., [26, 30, 43, 48, 61]). *The first section* aimed to gather information regarding the background of the participants: gender, age, university and the role at the university, the scientific domain and if the respondent is teaching or not climate change issues at the university. *The second section* of the survey, being devoted to the staff members in a university who integrated climate change-related aspects in a course, aimed to gather information on the perceived level of training of the teacher: climate change topics of the course, for which the teacher needs more training, the level of expertise to teach the course, how this was obtained and which sources of information are used to develop the content of the course. The item 14 of the questionnaire was composed of 13 statements assessing the perceived level of efficiency of the most important means to promote CCE. The assessment was made using a Likert-type scale (1—strongly disagree, 2—disagree, 3—no opinion, 4—agree, 5—strongly agree). Statistical processing was performed employing a two-stage cluster analysis to classify attitudes of respondents towards these statements. In this respect, a solution with two clusters was chosen using country/continent from where the respondents/university come from, their gender, age, staff role at the university and frequency of sciences as categorical variables. The difference between the two clusters was determined using the Mann–Whitney U test (*p* < 0.05).

The third section investigated the challenges and drivers of implementing climate change-related initiatives at the university level, aiming to assess the drivers and their potential. Two open-ended questions gave the possibility to the respondent to further emphasize through deeper understanding how the university could address the training needs on CCE. The validity of the data is further assured as it derived from bona fide academic institutions and supplied by well-informed sources. The reliability of data is also assured, since those who replied are very familiar with the concept of sustainability and have an understanding of the emphasis to this topic in their own institutions.

### Case-studies selection

In order to reveal how climate change is implemented by universities, mainly through teaching and research, a literature review has been performed. In parallel with covering climate change in bachelors/masters/PhD curriculum, they cover this topic through the following approaches: (i) by delivering different training events; (ii) by means of various public initiatives including workshops and conferences; (iii) by conducting specific research activities.

The selection of 12 universities as case studies was based on two criteria—ranking and geographic location. Criteria #1 was based on Academic Ranking of World Universities 2020 (http://www.shanghairanking.com/index.html), three universities from top 100, four universities from ranked between 101 and 1000; five universities out of list. Criteria #2 was based on the geographic location of universities: five from Europe; three from North America; four from other parts of the world.

## Results and discussion

### a. The results of bibliometric analysis

Results of the term co-occurrence analysis using VOSviewer allow us to identify what issues related to CCE have received more attention in the literature. These results (Fig. 2) show that, in addition to the search terms (i.e., climate change, education, universities, HE, and curriculum), terms such as adaptation, perceptions, science, management, policy, and several terms related to mitigation have received more attention. In other words, these terms have occurred more frequently in the reviewed literature, indicating that issues related to them have been more studied. Better understanding can be achieved by looking into the five major thematic clusters identified by the analysis that are shown in different colors in Fig. 2. These clusters show the major thematic focus of the existing literature and are, in descending order of importance, focused on climate change adaptation, sustainability and climate change mitigation, institutional aspects, challenges and barriers, and curriculum reform.

In the largest cluster (in red) terms related to climate change adaptation are dominant. This indicates the recognition of the significance of integrating knowledge about climate change adaptation in university education. In fact, communicating the potential risks posed by climate change is critical and has been well recognized in the literature [42]. It is believed that risk communication, overcoming misconceptions, and enhancing knowledge regarding adaptation strategies can contribute to better response to climatic threats in the long run [3, 42].

The second largest cluster (in green) is dominated by terms related to sustainability and climate change mitigation. It is increasingly recognized that CCE should be an integral part of efforts aimed at ESD [2, 38, 45]. Considering the central position of mitigation in climate change efforts and policies, and since the energy and water sectors are major contributors to climate change, there has been increasing attention to issues related to their efficient management [28]. The emphasis on the climate–energy nexus has highlighted the need for responsible consumption, and extension of efficient energy systems based on renewable energy technologies [28]. It is demonstrated that enhancing mitigation knowledge through CCE programs can make significant contributions to reducing lifetime emissions of individuals [13]. This cluster also includes the term “water resources” that, considering the water–energy nexus, may indicate the importance of appropriate and efficient water resource management for achieving climate change mitigation targets [49]. Water resource management is also essential for climate change adaptation.

Results also highlight the significance of CCE for better community engagement and for facilitating institutional transformations (blue cluster). Also, in some cases, institutional reform could be necessary for mainstreaming CCE in university programs. CCE is likely to increase community engagement in mitigation and adaptation efforts and promote pro-environmental attitudes and behaviors [7, 42]. Mainstreaming CCE may, however, be challenging as some institutions may resist change [7]. This highlights the need for institutional transition to facilitate integration of CCE into university programs [32]. Such institutional reforms and transitions can also facilitate CCE by developing strategic education programs and providing training programs to enhance competency of teaching staff [1, 29]. Institutional support in terms of, among other things, reforming regulations and fulfilling meeting requirements is also critical for addressing other challenges that may make it difficult to mainstream CCE into universities ‘education agenda.

The purple cluster in Fig. 2 highlights some of these barriers. One major challenge is that, traditionally, there has been a tendency towards disciplinary and silo-based education in universities. This is not conducive to CCE that requires inter- and trans-disciplinary approaches and collaboration between different fields and with various stakeholders [20, 29]. In this regard, institutional support through development of platforms for engagement and collaboration of stakeholders from different fields and/or offering incentives for participating in collaborative and interdisciplinary programs can be effective [1, 5, 29, 46]. In addition, as in the case of education for sustainable development (ESD), lack of experiences could be a reason for limited integration of CCE [46]. Accordingly, developing platforms for sharing successful experiences is necessary.

Finally, the presence of terms such as curriculum and students in the last cluster (yellow) is also noteworthy. Curriculum reform is essential since it may not be easy to integrate topics related to climate change into curricula that are traditionally developed along disciplinary lines [20]. Successful reform of curricula may, however, be challenging as not all students/teachers may favor it [25]. Therefore, overcoming such challenges is also essential.

### b. Results of the survey

**Fig. 3:**
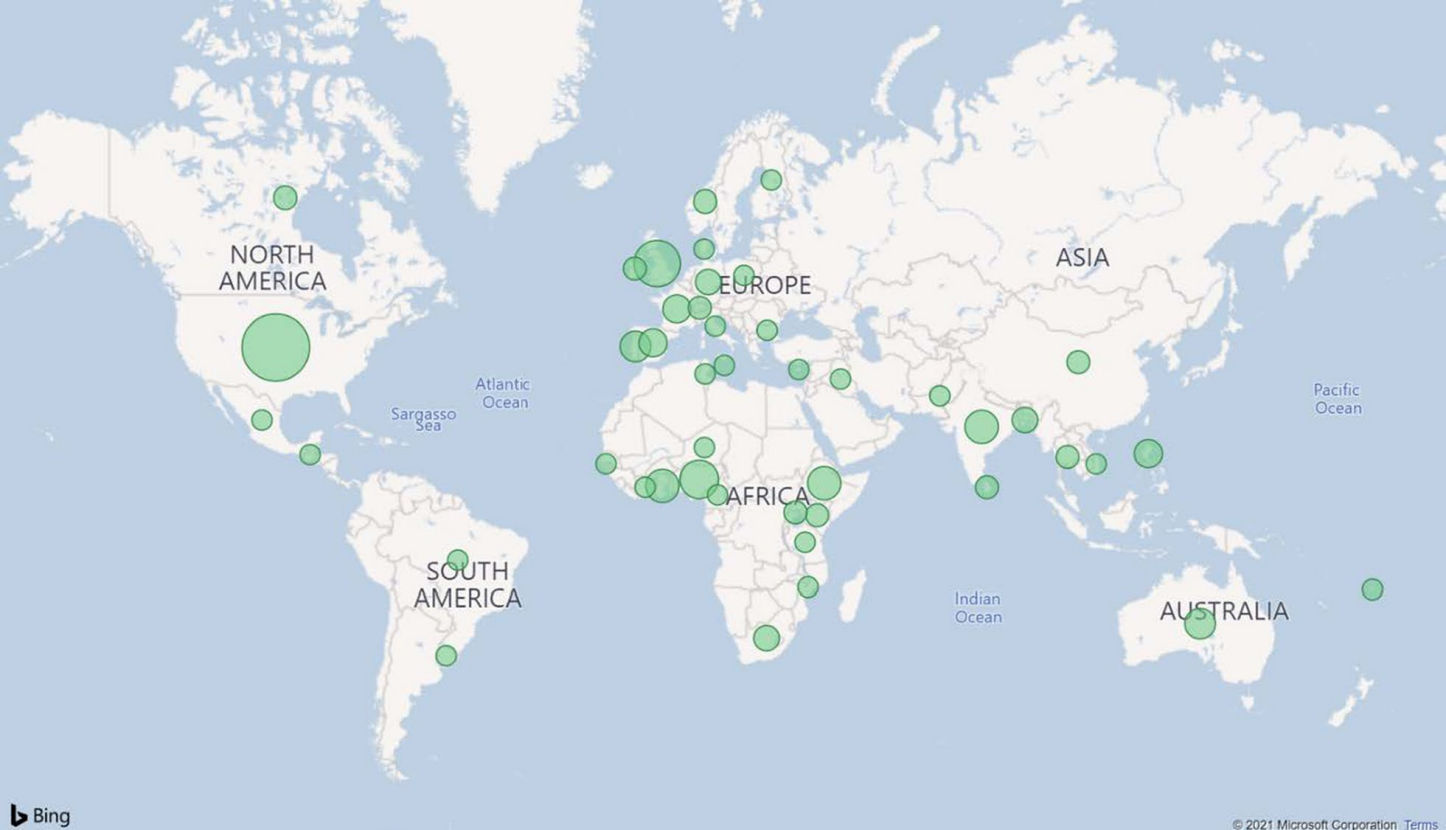
Participating countries in the survey

A total of 45 developed and developing countries represent the sample of the survey: Australia (3.9%), Bangladesh (2.4%), Brazil (0.8%), Bulgaria (0.8%), Cameroon (0.8%), Canada (1.6%), China (1.6%), Côte d'Ivoire (0.8%), Cyprus (0.8%), Denmark (0.8%), Ethiopia (4.7%), Fiji (0.8%), Finland (0.8%), France (3.1%), Gambia (0.8%), Germany (2.4%), Ghana (4.7%), Guatemala (0.8%), India (4.7%), Iraq (0.8%), Ireland (1.6%), Italy (0.8%), Kenya (1.6%), Malta (0.8%), Mexico (0.8%), Mozambique (0.8%), Niger (0.8%), Nigeria (6.3%), Norway (1.6%), Pakistan (0.8%), Philippines (3.1%), Poland (0.8%), Portugal (3.9%), South Africa (2.4%), Spain (3.1%), Sri Lanka (1.6%), Switzerland (1.6%), Tanzania (0.8%), Thailand (1.6%), Tunisia (0.8%), Uganda (1.6%), United Kingdom (8.7%), United States (15.0%), Uruguay (0.8%), Vietnam (0.8%). Figure 3 presents the worldwide distribution of responses.

Table 2 presents the sample characteristics. Most participants were male (63.6%), and the dominant knowledge areas were Social Sciences (31.8%) and Environmental and Earth Sciences (28.7%). Almost all age groups had rather balanced participation (between 20 and 27%), except for the youngest group (21 to 30 years old), which had the lowest participation. In terms of role, the permanent positions mainly involved in postgraduate and undergraduate teaching and research were the main ones indicated by the sample (40.3% and 24.8%, respectively). Regarding the university profile and institutional involvement in external climate change projects, research was the most important type of involvement observed (76.7%). Lower levels of responses were provided for community-related programs (45.0%) and teaching/training programs (36.4%). Other types of involvement mentioned were consultancy projects and events open to the public. No involvement was reported by 12.4% of the respondents, and even when the engagement was reported, they generally tend to occur to a moderate extent (41.9%) (*M* = 2.9520, SD = 0.99).

**Table 2:** Sample characteristics

Gender	%	Age (years old)	%
Male	63.6	21 to 30	3.1
Female	36.4	31 to 40	20.2
**Country classification**	%	41 to 50	27.1
Developed country	51.2	51 to 60	26.4
Developing country	48.8	more than 60 years old	23.3

Knowledge area	%	Role	%
Social Sciences	31.8	Permanent member of staff mainly involved in postgraduate teaching and research	40.3
Environmental and Earth Sciences	28.7	Permanent member of staff mainly involved in undergraduate teaching and research	24.8
Agrarian Sciences	8.5	Temporary member of staff	17.1
Engineering	7.0	Permanent member of staff mainly involved in research	14.0
Business studies	4.7	Permanent member of staff mainly involved in teaching	3.9
Biological Sciences	2.3		
Humanities/Linguistics	0.8		
Physical Sciences	0.8		
Other^a^	15.5		

^a^Other include: Agricultural Economics; Agroforestry and Forestry; Architecture and Urban Studies; Climate and Society; Climate Variability and Change; Coping/Adaptation Strategies, Modeling; Consulting; Education; Education for Sustainable Development; Energy science; Entrepreneurship Education; Environmental and Resource Economics; Environmental Economics; Environmental Health Science; Environmental Law; Environmental Sociology; Environmental studies (not science; studies in interdisciplinary); Student's Union; Sustainable Development and Business; Urban Planning

When asked if they teach climate change-related aspects in a course at the university, 77.5% responded positively (Additional file 2: Appendix S2 presents the list of courses taught as indicated by the sample). On the other hand, only 58.1% of the respondents indicated that climate change-related aspects are included in the course guidelines. The others either do not teach the topic (16.3%) or include these aspects in the teaching, even though not having the course guidelines (24.8%).

Table 3 presents a comparison between aspects of climate change mostly addressed by the courses taught by the sample and their training needs. The same list of aspects was presented and assessed in two different questions. As expected, the aspects of climate change mitigation and adaptation, and social and environmental impacts were addressed mainly by the courses, while the lowest rates were observed for ESG (Environmental, Social and Governance) reporting, climate diplomacy and climate leadership. In terms of more training needed by the educators, around one third of the sample indicated topics as projections of future climate change, the economics of climate change and climate governance. The least indicated topics include climate change mitigation and SDG 13.

**Table 3:**
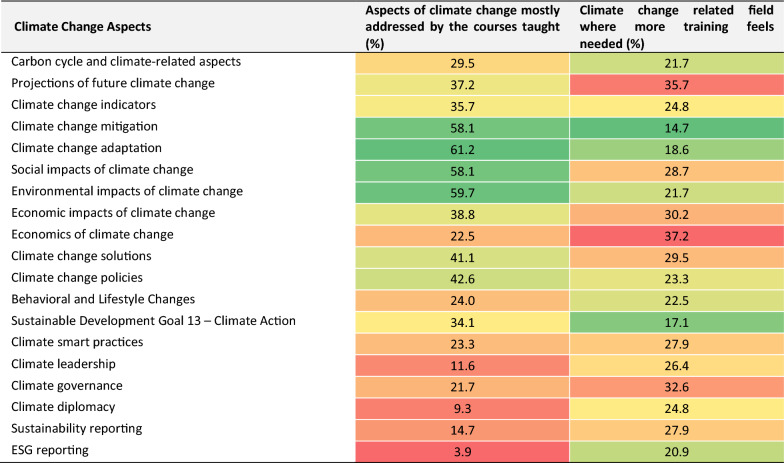
Comparison between aspects of climate change mostly addressed by the courses taught by the sample and their training needs

Other aspects listed by the sample in the additional open option as already being included in teaching cover a wide range of perspectives and topics: carbon trading, carbon footprinting, greenhouse gas inventorying, climate change impacts, climate and gender, climate change communication, climate finance, climate justice, leadership in sustainability, among others. In terms of the areas where the respondents need further training, the listed topics include: communication strategy, health impacts, history of climate change, how to educate in response to climate change, justice and equity impacts of climate change, keep on track with various information bases, public engagement, countering climate change denialism.

**Fig. 4:**
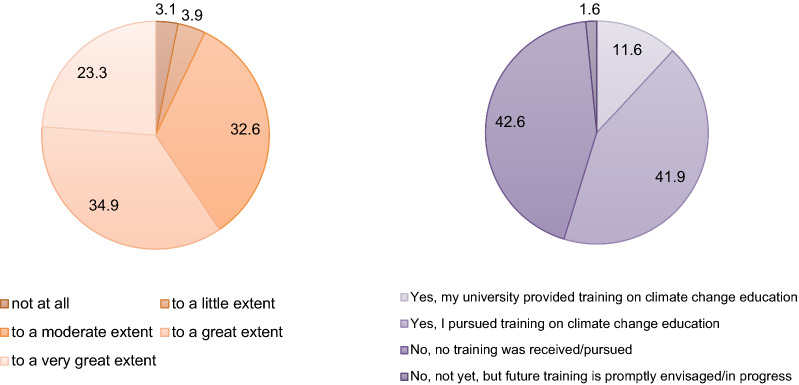
Sample perceived preparation to teach climate change-related concepts (*left*) and received/pursued training on climate change (*right*) (in percentage of responses)

Figure 4 presents the responses for two of the survey questions: “Do you feel prepared to teach climate change-related concepts?” and “Have you received or pursued training on matters related to climate change?”. More than half of the participants indicated that they feel prepared to teach the topic to a great or very great extent. Similarly, around 53% of the respondents said they already received or pursued training on climate change.

The questionnaire also focused on the primary sources the academic staff rely on to develop the content of courses. Scientific articles were the most indicated option (85.5%), followed by Internet-based resources (69.8%), Intergovernmental Panel on Climate Change (IPCC) Assessment reports (68.2%), own research (62.0%) and reports elaborated by other global organizations (58.1%). Printed books were also in the list (39.5%). Almost 10% of the respondents used the additional option to include other sources, such as national assessments of climate change projections and impacts, findings from other project research, the course textbook, workshops, conferences, and webinars.

Table 4 points out the efficiency of the means to promote CCE as stated by the respondents. The highest mean is observed for problem-based learning, followed by experiential learning and fieldwork. Online courses received the lowest mean, indicating probably the need for more developments in this area to support efficiency towards teaching and learning. In courses on climate change, the sample supported the need for placing specific emphasis on problem-based learning (48.1%), behavior change (37.2%), nature-based learning (36.4%), experiential learning (36.4%), cognitive learning (30.2%), and Socio-emotional aspects (30.2%).

**Table 4:** Perceived efficiency of the most important means to promote CCE (1—not efficient, 2—somewhat efficient, 3—efficient, 4—very efficient, 5—extremely efficient) (*N* = number, *M* = mean, *SD* = standard deviation)

	*N*	*M*	SD
Problem-based learning	125	4.20	0.93
Experiential learning (internships, residencies)	124	3.89	1.01
Fieldwork	123	3.82	1.03
Short-term training workshops	125	3.62	1.01
Didactic teaching	125	3.31	1.17
Online courses	123	2.93	1.03

When focusing on climate change and several aspects in the training process involving the universities, the academic staff generally showed a high level of agreement with the presented statements (Table 5). Based on the Likert scale used (from 1 'strongly disagree' to 5 'strongly agree’), respondents in both identified clusters agree that universities should offer space for climate change education, that climate change is a real concern for the country and that there is a growing demand for experts and professionals in climate change (Table 5, scores above 4). On the other side, respondents have a neutral opinion with the statements referring that the university has properly embedded climate change into teaching and learning activities and offers nature-based/nature-immersive courses to cultivate care for the environment (Table 5, scores around 3). Finally, the sample of respondents does not agree whether there is lot of skepticism related to climate change among students, as this topic had the lowest agreement mean (Table 5, scores below 3).

**Table 5:** Description of the two clusters in terms of country/continent, gender, age, staff role and knowledge area (*N* = 129)

Demographic characteristics	Cluster #1 (*N* = 72)	Cluster #2 (*N* = 57)	Total (*N* = 129)
**Continent**			
Europe	18 (46.2%)	21 (53.8%)	39 (100%)
Asia	13 (59.1%)	9 (40.9%)	22 (100%)
Africa	21 (55.3%)	17 (44.7%)	38 (100%)
Americas and Australia	20 (66.7%)	10 (33.3%)	30 (100%)
**Gender**			
Male	47 (57.3%)	35 (42.7%)	82 (100%)
Female	25 (53.2%)	22 (46.8%)	47 (100%)
**Age**			
Below 50 years of age	33 (50.8%)	32 (49.2%)	65 (100%)
Above 50 years of age	39 (60.9%)	25 (39.1%)	64 (100%)
**Staff role**			
Teaching and research	53 (63.1%)	31 (36.9%)	84 (100%)
Only teaching or only research	5 (21.7%)	18 (78.3%)	23 (100%)
Temporary staff	14 (63.6%)	8 (36.4%)	22 (100%)
**Sciences**			
Environmental sciences	22 (59.5%)	15 (40.5%)	37 (100%)
Engineering and life sciences	11 (55%)	9 (45%)	20 (100%)
Social sciences	26 (54.2%)	22 (45.8%)	48 (100%)
Natural and other sciences	13 (54.2%)	11 (45.8%)	24 (100%)

Level of agreement with climate change statements	M ± SD^1^	M ± SD^1^	M ± SD^1^
Climate change is a real concern for my country	4.7 ± 0.8	4.3 ± 1.1	4.5 ± 1.0
The university should offer space for CCE	4.8 ± 0.6	4.5 ± 1.0	4.6 ± 0.8
My university has properly embedded climate change into teaching and learning activities	3.6 ± 0.9^a^	2.4 ± 0.9^b^	3.1 ± 1.1
My university offers nature-based/nature-immersive courses to cultivate care for the environment	3.8 ± 0.8^a^	2.3 ± 0.9^b^	3.1 ± 1.1
There are interdisciplinary research units in my university to address climate change issues	4.2 ± 0.8^a^	2.4 ± 1.0^b^	3.4 ± 1.3
I am aware of an on-going program developed by my university in the field of climate change	4.3 ± 0.8^a^	2.2 ± 1.0^b^	3.4 ± 1.4
Students in my university are keen to receive training on climate change	4.2 ± 0.8^a^	3.2 ± 1.1^b^	3.8 ± 1.1
Students in my university change their belief in climate change throughout the course	3.6 ± 0.9^a^	3.0 ± 0.8^b^	3.4 ± 0.9
There is a lot of skepticism related to climate change among students in my university	2.3 ± 1.1	2.5 ± 1.0	2.4 ± 1.1
Students in my university are motivated to undertake climate initiatives and take action in everyday life	3.8 ± 0.7^a^	3.1 ± 0.9^b^	3.5 ± 0.9
Students can have a better career pathway if they have good climate change literacy	4.1 ± 0.9	3.7 ± 1.0	3.9 ± 1.0
There is a growing demand for experts and professionals in climate change in my country	4.2 ± 0.9^a^	3.6 ± 1.1^b^	4.0 ± 1.0
Climate change training in my university will increase in the future	4.2 ± 0.7^a^	3.3 ± 0.9^b^	3.8 ± 0.9

The mean ± standard deviations (M ± SD) values^1^ were obtained from the raw data. Note: Items denoted with different letters are significantly different at the level of 5%

Likert scale: (1) “Strongly disagree”, (2) “Disagree”, (3) “No opinion”, (4) “Agree”, (5) “Strongly agree”

The cluster analysis revealed two clusters, based on the scores presented in Table 5, that can be identified as “climate active universities” and “less climate active universities”. The first cluster mainly consists of respondents/universities from Asia, Africa, Americas and Australia and some western and Northern European countries, of higher ages (above 50 years of age), mainly engaged in teaching and research. Opposed to them, the demographic structure of “less climate active universities” are mainly from Europe (mainly from Central, Eastern and Southern Europe) and some institutions in Africa, with an age below 50 and respondents that are engaged either only as teachers or only as researchers. Out of 13 statements, for nine there are statistically significant differences between the clusters (*p* < 0.05, denoted with different letters in Table 5).

While the respondents in both clusters scored almost equally for the first two statements (climate change is a real concern for the country and that the university should offer space for CC, *p* > 0.05), the first group of “climate active universities” showed a higher agreement (scores around 4) with the statements referring to the awareness in terms of climate-related initiatives (research units, programs) undertaken by the university, but also with the statements that there is a growing demand for experts and professionals in climate change, thus climate change is likely to increase in the future and to the fact that students are keen to receive training on CC. For the same statements, there is a low agreement (scores around 2) for the group named “less climate active universities”. Both clusters express the same level of disagreement associated with skepticism related to climate change among students (*p* > 0.05) and the same level of agreement that climate change literacy enables better career pathways (*p* > 0.05).

**Fig. 5:**
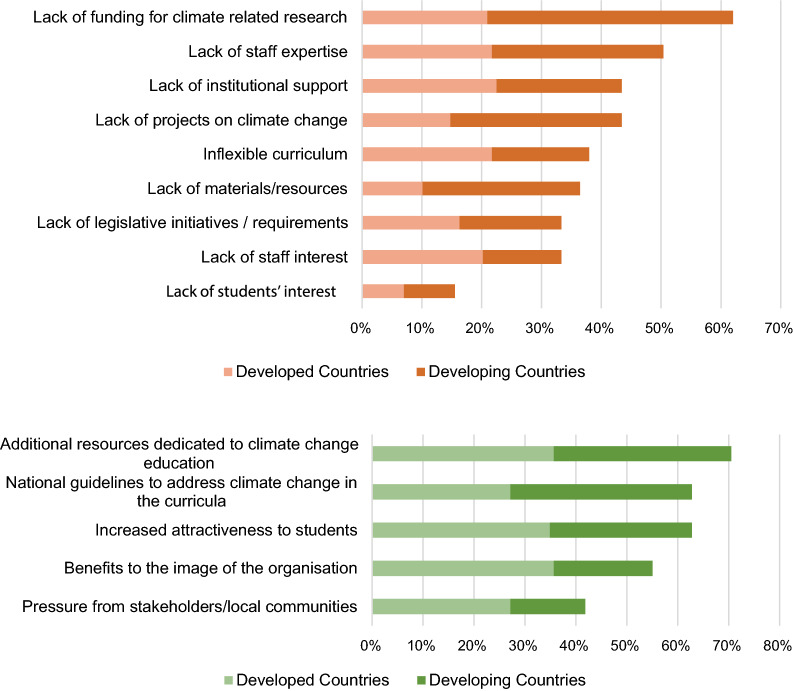
Challenges (*above*) and Drivers (*below*) to implementing CCE at the respondents ‘ universities (in percentage of responses)

As far as challenges and drivers to implementing CCE are concerned, Fig. 5 summarizes the sample views. Lack of funding for climate-related research was the most indicated challenge (by roughly 63% of the sample—of this number, two-thirds from developing countries). Lack of staff expertise had also a high response (over 50%), supporting the need for further climate change training. On the other hand, lack of students’ interest does not seem to be a considerable challenge (15.5%). Other challenges mentioned by the sample (11%) in the open space consist of lack of internal resources for scientific development, lack of leadership from highest campus officials, lack of progression between climate-related courses, lack of diversity in faculty expertise, lack of targeted on-job short to medium term trainings on climate change and related issues, lack of internship opportunities for students to develop their practical skills on climate change solutions, lack of will to pursue CCE against all odds, and religious or political perspectives.

As for drivers, additional resources dedicated to CCE (selected by 71.3% of the sample) were perceived as fundamental for improvements in this area, followed by national guidelines to address climate change in the curricula (63.6%) and the increased attractiveness to students (62.8%). Pressure from stakeholders and/or local communities does not seem to be an essential driver, as it was indicated by around half of the respondents. Other drivers were indicated by less than 7% of the respondents, and include being relevant in global discourse on climate issues, competition among universities in terms of courses offered to their graduates, increased job opportunities directly arising from climate change concerns in businesses and communities, organized faculty support, personal motivation, potential national and international collaborations with research institutions and academic institutes, as well as any non-governmental organizations, and the existence of local (e.g., state) guidelines to address climate change in the curricula.

The last survey questions referred to the perceived potential for CCE in the following years and how it could be addressed. In general, the sample saw potential on the topic (*M* = 3.8281; SD = 0.89715), with over 90% of the respondents spread around the positive response options (26.4% to a moderate extent, 43.4% to a great extent, and 23.3% to a very great extent). A total of 93 respondents (out of 129) used the open space (response was not mandatory) to describe how training needs on CCE can be addressed at the university level.

**Fig. 6:**
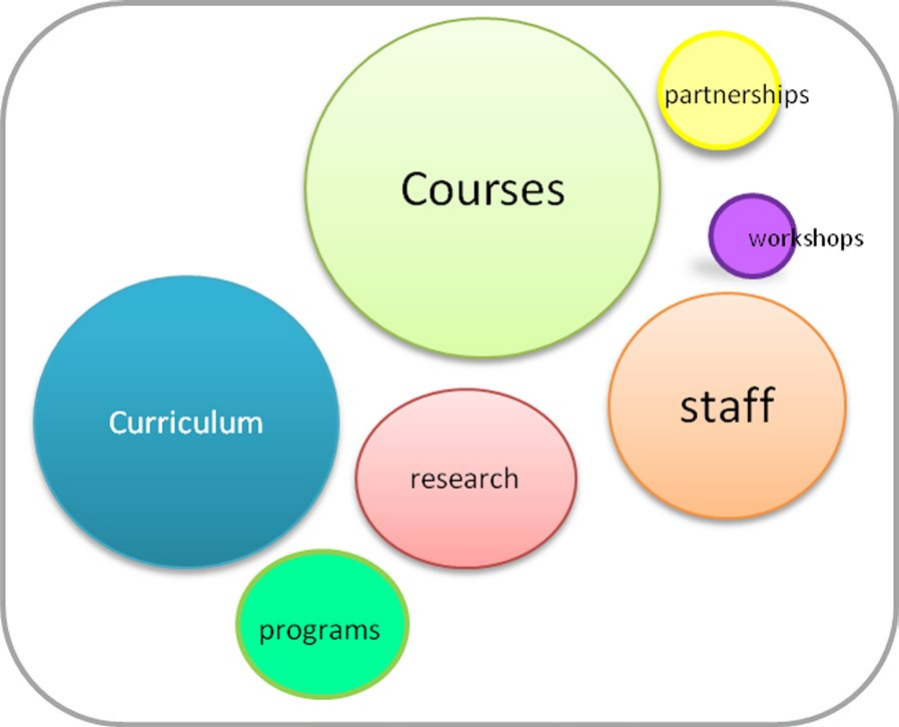
The major themes for training needs in terms of CCE at universities identified by the participants in the survey

By coding the responses against the major themes, several aspects were identified as having the highest frequency (proportionally represented in Fig. 6), being the significant needs in terms of climate change at universities. The main one identified by the respondents is related to the courses, either by introducing climate change aspects in the existing courses, or designing new interdisciplinary courses. Even the importance of online courses was raised in this respect. Another main need was the curriculum in terms of introducing a flexible and interdisciplinary curriculum, embedding deeply the climate change aspects and allocating more time for them, following by the need to increase staff training and capacity. With a lower frequency, research in terms of funding projects related to climate change was mentioned by the respondents, but also the need to enhance the partnership, collaboration and exchange between the universities, to design specialized university programs, and to organize various events as workshops, seminars and conferences. Some respondents also mentioned community outreach activities, the involvement of students and creating special departments on climate change as being relevant actions to address the climate change needs at universities.

These identified needs are also the main instruments used by the 12 selected universities analyzed in the paper to develop climate change actions. The results of the survey are also in line with the challenges to CCE identified in the literature review part of the paper: the strong need identified in the previous publication for institutional support to integrate CCE into the university programs [32], but also the curriculum reform to overcome the disciplinary perspective by introducing interdisciplinary courses [20]. This emphazizes the role of the institutional support to CCE by developing specialized training programs and reforming the curricula, raising the competencies of teaching staff being another theme identified in the literature [1, 29]. This is in line with the strong need perceived by the respondents of the survey regarding staff training and increasing the capacity of staff to teach the climate change aspects along various courses.

### c. Case-studies analysis

Table 6 summarizes the approach used by 12 universities around the world in terms of developing climate-related actions. It is obvious that development of at least one curriculum at one of the three levels of studies is mostly the first step. Also, universities within their different research projects and activities produce graduate theses (mostly PhDs) that have climate change in focus. As a result of these research activities, universities also organize conferences/workshops and have a growing publication record dedicated to climate change.

**Table 6:** Examples of means used by 12 universities around the world in terms of developing climate-related actions

University	Case study title	Approach used	Impact	References
Hamburg University of Applied Sciences	Research and Technology Transfer Sustainability and Climate Change Management	Training on climate change Editing book series on Climate change	Over 3.000 academic staff Over 15 books	[17, 22]
University of British Columbia	Climate Action Plan 2030—CAP 2030; and research	Engagement of staff and students in the actions conducted in the Climate Action Plan 2030—CAP 2030, various post-graduate theses	Staff and students	[55, 66]
University of Toronto	Climate Change Policy and Practice	Life learning program course	Students and participants in life-learning program	[66]
University of Campinas	Diversified actions	Courses in undergraduate and graduate programs, research (various post-graduate theses, research center)	Students, researchers	[9, 63]
University of Colombo	Diversified actions	Courses, postgraduate program on 'Climate Change and Environmental Management', campus management (waste management and carbon footprint reduction), various undergraduate and post-graduate theses, conferences	University community including students, public	[64]
Nottingham Trent University	Carbon Literacy Training' and online course 'Sustainability in Practice (SiP) Certificate'	Training; co-curricular online module	250 students and 120 staff; SiP offered to all 34,000 students online, 6233 completions to date	[44]
KTH Royal Institute of Technology	Diversified actions	Engagement of staff and training of academic staff (to integrate sustainability into education). Climate education integrated in all educational programs	Staff and students Divestment towards stakeholders with anti-climate change activities	[31]
Massachusetts Institute of Technology	Fossil Fuel divestment days	Divestment	Administration and students	[10, 37]
University of Latvia	Diversified actions	Courses, various post-graduate theses, conference (in progress)	More than 500 students in one of the courses, researchers Working on climate-change legislative	[65]
University Fernando Pessoa	Diversified actions	PhD program in Earth Sciences, research, various post-graduate theses, and conferences	Students and researchers	[59]
University of Fort Hare	Research center 'Risk & Vulnerability Science Centre'	Research, workshops, various post-graduate theses	Researchers and rural and local communities	[58]
Indian Institute of Technology Roorkee	Research on SDGs (20 thesis focusing on climate change)	Research, various post-graduate theses	Researchers	[27]

In parallel with teaching and research activities, some universities observe climate change from a wider perspective, providing voluntary training for their staff and students, life-learning and other types of courses to the public, or organizing public activities raising awareness to climate change challenges. In this context, the cases presented in this section have been grouped based on how the selected universities have been addressing the needs for increased and advanced climate change education, as identified in the previous methodological step: (i) accredited teaching programs of all levels of studies and research activities; (ii) various training events focused on staff and/or students; (iii) public initiatives; and (iv) other climate change-related activities.

### Accredited teaching programs of all levels of studies and research activities

One of the goals at KTH Royal Institute of Technology [31] is that all educational programs at all levels should integrated climate action in their curricula so that students after graduation can contribute to achievement of a climate neutral society. The university has an active collaboration with the KTH Student Union and student associations to support them in their work to monitor issues that concern sustainability and climate, among others, within education. Staff in leading teaching roles, such as the Director of First and Second Cycle Education, the Director of Third Cycle Education, program coordinators and individuals on the education committee and the equivalent should undergo training in how sustainable development (including climate issues) can be integrated in educational programs. Currently, in the first, second and third cycle, there are educational programs that have a focus on sustainable development and climate transformation.

At the University of Campinas, there are courses in undergraduate and graduate programs focusing on climate change issues (Geographic Climatology; Climate changes; Global Ecology and Climate Change). Among the topics addressed in these courses, it can be mentioned: effects of climate change on plants, analysis of climate changes, and to analyze at global scale the planet’s system, predicting and understanding environmental changes [63]. At the University, there are 100 documents presented in the online academic repository focusing on climate change. From them, 11 are PhD thesis and 35 are master dissertation [63]. Besides, the university has the Center for Meteorological and Climate Research Applied to Agriculture (CEPAGRI). Founded in 1983, the Center performs research on agroclimatology, agrometeorology, ecophysiology, and geotechnologies and provide several information about these themes for population. Studies on climate change are an important focus of CEPAGRI [9].

In the University of British Columbia (Canada) repository, 85 thesis and dissertations are focused on climate change issues, with 36 PhD thesis and 49 master dissertations. Among these documents, the oldest one was published in 1994: a master dissertation about sea level rising. In 2021, the two PhD thesis published (until April, 2021) were related to the impact of climate change on fish stock/supply [56].

Starting from 2012, the University of Latvia [65] has been engaged on climate change and sustainable development which has been advanced since 2014 by the development of a study course "Climate change and sustainable development" offered as elective with over 500 students having these lectures. Since 2018 another course is delivered, entitled "Management of Climate Change". At this institution, over 20 BSC, 8 MSc and one PhD theses have analyzed climate change. Impact of such an initiative has been observed by inviting academics to participate in drafting "Climate Law" (in progress) and National climate policy. In 2021, an international conference on Climate change in teacher education is planned.

Climate change is being addressed at the University Fernando Pessoa [59] within its PhD program in Earth Sciences, comprising the research branches, Oil Systems and Energy Problems, and Geo-risks, Gas Emissions and Geological Sequestration of CO2, through a specialized Isotherm Laboratory (measuring the capacity of coal as non-conventional reservoir to safety store CO2) to study CO2 sequestration in coal, aiming to contribute to Climate Change minimization. In 2008, a Workshop on “Energy, Greenhouse Gases and Environment” was held in Porto. Actions are being developed since the PhD program in Earth Sciences was officially implemented, in 2009. Until now, four PhD theses associated with climate change have been defended, and 12 peer-reviewed publications.

Similar can be seen at University of Colombo [64] with climate change courses for students at various levels of studies (Climate Change, Adapting businesses for climate change) and a postgraduate program on 'Climate Change and Environmental Management'. This University was the hosting partner of the International Climate Change Conference 2017 and 2018. Over 500 students have been included in various climate change lectures, with 1 PhD and 13 MSc/BSc (Honors) theses defended, and 12 articles related to climate change published in peer-reviewed journals within the last 5 years.

Within the Indian Institute of Technology Roorkee [27], specific research has targeted climate vulnerability based on land use, soil and climate, temperature and rainfall with urban civic labs incorporating relevant SDGs. Outcome comprises over 20 theses from different levels of studies covering climate change with several publications.

Within its curriculum, Hamburg University of Applied Sciences covers climate change as a topic included in Bachelors and Masters degree programs [22]. Within the scientific community, research group at Hamburg University of Applied Sciences is editing the world's leading peer-reviewed book series on climate change (the 'Climate Change Management' series) published by Springer which has produced various volumes such as 'Water, Energy and Food Nexus in the Context of Strategies for Climate Change Mitigation', 'Climate Change and the Role of Education or Climate Change, Hazards and Adaptation Options', among others.

### Various training events focused on staff and/or students

Hamburg University of Applied Sciences is an example of successful delivering training on climate change for over 3.000 academic staff [17]. It was organized by the research group “Research and Technology Transfer Sustainability and Climate Change Management” which launched in 2021 a new research on UN SDG13 “The Climate Change and SDG 13 Academic Research and Publications Initiative” [22].

Ranked in the first position of the [51] Ranking regarding universities activities related to climate action (SDG 13) [52], the University of British Columbia (Canada) established a plan to address climate change through actions within their campuses (Climate Action Plan 2030-CAP 2030). Through this plan, the university is engaging its staff and students in the process, providing a channel for them to ask questions, give feedback and actively engage in the actions conducted [55].

Nottingham Trent University [44] has run a 'Carbon Literacy Training' supporting staff and students with carbon literacy. It is an offer by the Green Academy at Nottingham Trent University that designed this Climate Education Course and in parallel designed one of the two open-source Carbon Literacy Toolkits for Higher Education (HE) and Universities funded by BEIS for the HE sector in the UK [41]. In total 13 internal courses have been organized in the last 2 years, with over 250 students and 120 staff participating. Also, this university has offered an online course 'Sustainability in Practice Certificate' (SiP) to all 34.000 students at NTU since 2013, covering all SDGs with a stronger focus on SDG13 added in 2019; 6233 students have completed SiP to date.

In December 2019, KTH Royal Institute of Technology [31] decided on university-wide climate goals for education, research, collaboration and campus activities. As one of initiatives—all employees at KTH need to have knowledge of climate challenges and actively work to reduce the climate impact based on their role and function at KTH.

### Public initiatives

Nottingham Business School, a faculty within Nottingham Trent University, has led the design and distribution of the 'Carbon Literacy Training for Business Schools', which over 400 Academics representing more than 100 business schools in 42 countries worldwide have completed successfully during 2019 and 2020 [50].

At University of Colombo [64], awareness programs on climate change have also been conducted for school children, employees at certain institutions, and public (i.e., through public lectures). At campus, awareness is raised by reducing carbon footprint over time, and better waste management.

As a result of an action initiated by administration and students at MIT (Massachusetts Institute of Technology) associated with the impact of fossil fuels on climate change [10], this institution is divesting by the endowment from companies that are developing fossil fuel resources, spreading various types of climate change disinformation and performing anti-climate lobbying by promoting their divestment and organizing Fossil Fuel divestment days [37].

University of Toronto within its School of Continuing studies (part of a life-learning program) is another example since they launched the course 'Climate Change Policy and Practice' associated with learning the methodology for calculating GHG emissions, GHG reporting and risk management [66].

The COVID-19 pandemic has provided an additional impulse to new events and activities at universities [19].

### Other climate change-related activities

The University of Fort Hare [58] has a research center 'Risk & Vulnerability Science Centre' strategically established in 2011 by South Africa’s Department of Science & Innovation. Its main goal is to cope and adapt capacities of rural communities to climate change by improving scientific understanding and developing technologies and innovations to respond to challenges induced by climate change. As a scientific center it has an annual budget to conduct climate change research and since its inception, the budget has been increasing every year. Due to the climate change catastrophes (droughts and floods) experienced in South Africa with more effects being felt within the municipality since 2019, the Centre has performed research collaborations from external local and international funders to tackle climate change issues facing the province. The Centre was selected to conduct climate change awareness workshops for the 2019 National Science Week by the South African Agency for Science and Technology Advancement (SAASTA), entitled 'Facing the Harsh Realities of Climate Change'. It also trained 60 unemployed youths on Household Food Security (including growing of climate resilient crops) in the face of climate change through a short learning program, assisted 15 unemployed youths to establish and register 2 cooperatives on crop production of climate resilient crops in South Africa and trained 25 unemployed youths on climate risks and environmental education.

The University of Colombo [64] applied for the Green Metric award in 2020, and was ranked fourth among the other universities in the country, however, the University of Colombo earned the highest marks for the Energy and Climate Change category. Currently, several climate change-related research projects involving local and international collaborators are underway.

## Conclusions

This paper has presented an overview of the extent to which matters related to climate change are tackled within the teaching and research practices at universities, with a focus on the training needs. There is a perceived need for this climate change-related research, since climate change is a major global problem, and knowledge about it is becoming increasingly important for future professionals, who need to be made familiar with strategies for its mitigation and adaptation as part of their university studies. Through a mixed research strategy, which entailed a bibliometric research, a worldwide online survey and case studies, the study shed some light on various aspects related to teaching and further needs on climate change within university programs.

In general, considering the great diversity of results presented in section three, one of the conclusions that can be drawn is that due considerations to CCE in HE courses are perceived by universities as important, since strategies to promote mitigation and adaptation demand multidisciplinary approaches. Also, as highlighted in the survey results, respondents in general reported that in their countries climate change is perceived as a matter of genuine concern, acknowledging that there is a growing demand for professionals with training in this area.

It is a matter of fact that not all universities are fully prepared for addressing climate change in their curricula and further, thus improvements are needed. One of the areas to be improved is in respect of curriculum innovations, i.e., making provisions to include climate change in teaching programs, and in various courses across the spectrum of academic disciplines. To do so, some of the barriers identified in this paper need to be overcome, and one of such barriers is the limitation in the training of teaching staff. In addition, international partnerships for CCE promotion and more opportunities for exchange of experiences among institutions can be highlighted as one of the steps that should be taken, to facilitate curricular innovations.

This study has some limitations. For instance, in the bibliometric analysis, the studied documents were collected via the WoS database and analyzed using the VOSviewer software. Even though the WoS database is one of the most relevant scientific databases in the world, there are others that could have also been used, to offer a comparison. Regarding the VOSviewer software, the parameters used in the analysis, and chosen by the researchers had their scope limited to the main focus of the study, namely on levels of emphasis to CCE. Regarding the survey, the size sample is too small to allow a great generalization of the results. Finally, regarding the case studies, even though 12 of them were analyzed, a greater diversity could provide a wider range of results.

Despite these constraints, the study provides a welcome addition to the literature, since it offers an overview of the training needs related to sustainable development in HE. The sample of 45 countries offers a rough profile of the trends seen today, whereas the case studies illustrate some of the potentials seen in this rapidly growing field.

The implications of this paper and the research performed are threefold. Firstly, it reiterates the growing emphasis universities give to climate change, as documented in the literature. Secondly, it outlines the key role played by training provisions in this process. Thirdly, it showcases examples of successful inclusion of matters related to climate change in university programs, and how some of the challenges may be overcome. Based on the lessons learned, some of the actions universities may adopt to better take into account matters related to climate change are:

i. A cross-cutting emphasis to climate change, across courses and disciplines;
ii. Identification of specific strengths and weaknesses in the curriculum for further improvement;
iii. Greater provisions for training programs for academic staff, so as to encourage them for a greater engagement in this area;
iv. Build a bridge between climate change teaching and research to maximize the synergies.

As to future studies, it is suggested that further works documenting experiences of curricular innovations for CCE in HEIs in different regions are undertaken, especially in respect of achieving synergies among disciplines.

Due to the urgency of the climate challenges seen today—and expected in the future—this paper argues that no university can afford to ignore this topic. As seen in the results and discussion section, there are many on-going initiatives in respect of climate change teaching, which show it is perfectly feasible to engage in them. Teaching initiatives, combined with climate change research programmes, can make sure that universities are able to make their contribution towards addressing a problem, which is global in nature but whose impacts are mostly felt at the local level.

## Supplementary Information

**Additional file 1: Appendix S1.** Survey on training needs on climate change education at universities.

**Additional file 2: Appendix S2.** List of courses taught by the participants in the survey.

## Data Availability

The datasets used and/or analyzed during the current study are available from the corresponding author on reasonable request.
