# Supplementary material for: Handling climate change education at universities: an overview
Source: Environ Sci Eur. 2021 Sep 25;33(1):109. doi: 10.1186/s12302-021-00552-5 (PMC8475314; doi:10.1186/s12302-021-00552-5)
Supplement: Supplementary file 1 — Additional file 1: Appendix S1. Survey on training needs on climate change education at universities. [file 12302_2021_552_MOESM1_ESM.pdf]

Appendix S1. Survey on Training needs on climate change education at universities

Dear Colleagues,
This questionnaire is part of an international study undertaken by the European School of Sustainability Science and Research (ESSSR) https://esssr.eu/ and the International Sustainable Development Research Programme (IUSDRP) https://www.hawhamburg.de/ftz-nk/programme/iusdrp.html, aimed at identifying knowledge gaps on climate change education at universities.

We would grateful if you could answer the questions below, which take approximately 10 minutes. All individual answers will be treated with confidentiality, and no personal data is gathered or stored.

If you wish to receive a copy of the results, please state so, by using the box at the end of the questionnaire. Thank you.

Regards,
The ESSSR and IUSDRP Teams

Section 1 – Background

1. **Your role at the University:**

( ) Permanent member of staff mainly involved in undergraduate teaching and research
( ) Permanent member of staff mainly involved in postgraduate teaching and research
( ) Permanent member of staff mainly involved in teaching
( ) Permanent member of staff mainly involved in research
( ) Temporary member of staff
( ) Other: (please specify)

2. **Your gender:**

( ) Male
( ) Female
( ) Prefer not to say

3. **Country of your university:**

(add list of options as dropdown menu)

4. **Your age group:**

( ) 21-30
( ) 31-40
( ) 41-50
( ) 51-60
( ) More than 60 years old

( ) Prefer not to say

5. **In which scientific area do you teach? (multiple answers possible)**

( ) Environmental and Earth Sciences
( ) Biological Sciences
( ) Chemical Sciences
( ) Physical Sciences
( ) Mathematical Sciences
( ) Engineering
( ) Health Sciences
( ) Agrarian Sciences
( ) Social Sciences
( ) Humanities/Linguistics
( ) Business studies
( ) Other (please specify)

Section 2 – Your experience with Climate Change Education

6. **Do you currently teach climate change-related aspects in a course at the university?**

( ) Yes
( ) No (if no, the respondent should be directed to Section 3)

6.1 If Yes, could you please indicate the name of the course?

____

7. **Are climate change-related aspects included in the guidelines of the course or have you chosen to address them along with another topic (e.g. environmental impacts)?**

( ) Climate change-related aspects are included in the course guidelines
( ) Climate change-related aspects are not included in the course guidelines but I have included them in the teaching

8. **Which aspects of climate change are mostly addressed by the course that you teach? (multiple answers possible)**

( ) Carbon cycle and climate-related aspects
( ) Projections of future climate change
( ) Climate change indicators
( ) Climate change mitigation
( ) Climate change adaptation
( ) Social impacts of climate change
( ) Environmental impacts of climate change

( ) Economic impacts of climate change
( ) Economics of climate change
( ) Climate change solutions
( ) Climate change policies
( ) Behavioral and Lifestyle Changes
( ) Sustainable Development Goal 13 – Climate Action
( ) Climate smart practices
( ) Other: (please specify)

9. In which field related to climate change do you feel you need more training? (multiple answers possible)

( ) Carbon cycle and climate-related aspects
( ) Projections of future climate change
( ) Climate change indicators
( ) Climate change mitigation
( ) Climate change adaptation
( ) Social impacts of climate change
( ) Environmental impacts of climate change
( ) Economic impacts of climate change
( ) Economics of climate change
( ) Climate change solutions
( ) Climate change policies
( ) Behavioral and Lifestyle Changes
( ) Sustainable Development Goal 13 – Climate Action
( ) Climate smart practices
( ) Other: (please specify)

10. Do you feel prepared to teach climate change-related aspects?

( ) Not at all
( ) To a little extent
( ) To a moderate extent
( ) To a great extent
( ) To a very great extent

11. Have you received or pursued training on matters related to climate change?

( ) Yes, my university provided training on climate change education
( ) Yes, I pursued a training on climate change education
( ) No, no training was received/pursued
( ) No, not yet, but future training is promptly envisaged/in progress

12. **Which are the main sources you rely on to develop the content of your courses? (multiple answers possible)**

( ) Internet-based resources
( ) Scientific articles
( ) IPCC Assessment reports
( ) Reports elaborated by other global organisations
( ) Printed books
( ) My own research
( ) Other: (please specify)

13. **Please rate the perceived efficiency of the most important means to promote climate change education (1-not efficient, 2-somewhat efficient, 3-efficient, 4-very efficient, 5-extremely efficient)**

	**1**	**2**	**3**	**4**	**5**
Didactic teaching					
Short-term training workshops					
Experiential learning (internships, residencies)					
On-line courses					
Fieldwork					
Problem-based learning					
Others (please specify)					

14. **To what extent do you agree with the following statements? (1=fully disagree, 2= disagree, 3= not sure, 4=agree, 5=fully agree)**

	**1**	**2**	**3**	**4**	**5**
Climate change is a real concern for my country					
The university should offer space for climate change education					
My university incorporated properly climate change into teaching and learning activities					
My university offers nature-based / nature-immersive courses to cultivate care for the environment					
There are interdisciplinary research units in my university to address climate change issues					
I am aware of an on-going programme developed by my university in the field of climate change					
Students in my university are keen to receive training on climate change					

Students in my university change their belief in climate change throughout the course					
There is a lot of skepticism related to climate change among students in my university					
Students in my university are motivated to undertake climate initiatives and take action in everyday life					
Students can have a better career pathway if they have good climate change literacy					
There is a growing demand for experts and professionals in climate change in my country					
Climate change training in my university will increase in the future					

15. In the courses on climate change, specific emphasis should be placed on (multiple answers possible):

( ) Cognitive learning
( ) Socio-emotional aspects
( ) Nature-based learning
( ) Behavior change
( ) Experiential learning
( ) Problem-based learning
( ) A combination of all

Section 3 – Challenges and drivers

16. Which are the challenges to implementing climate change initiatives at your university? (multiple answers possible)

( ) Lack of staff expertise
( ) Lack of staff interest
( ) Lack of students' interest
( ) Inflexible curriculum
( ) Lack of materials/resources
( ) Lack of projects on climate change
( ) Lack of institutional support
( ) Lack of legislative initiatives / requirements
() Lack of funding for climate related research
( ) Other: (please specify)

17. Which are/could be the main drivers for implementing climate change education at your university? (multiple answers possible)

( ) National guidelines to address climate change in the curricula
( ) Benefits to the image of the organisation
( ) Increased attractiveness to students
( ) Pressure from stakeholders/local communities
Other: ________

18. Is your university involved in external climate change projects? If so, in which types? (multiple answers possible)

( ) Research
( ) Teaching/training programmes
( ) Community-related programmes
( ) Other: (please specify)

18.1If so, to which extent?

( ) Not at all
( ) To a little extent
( ) To a moderate extent
( ) To a great extent
( ) To a very great extent

19. As teaching staff, do you see an increased potential for climate change education in the next years?

( ) Not at all
( ) To a little extent
( ) To a moderate extent
( ) To a great extent
( ) To a very great extent

20. If so, how could universities address training needs on climate change education?

____
____
____
____

21. Are there any other aspects related to training needs on climate change education at universities that you consider important and that were not covered by this survey? (please explain)

____
____

Thank you for your participation!
