# Supplementary material for: Handling climate change education at universities: an overview
Source: Environ Sci Eur. 2021 Sep 25;33(1):109. doi: 10.1186/s12302-021-00552-5 (PMC8475314; doi:10.1186/s12302-021-00552-5)
Supplement: Supplementary file 2 — Additional file 2: Appendix S2. List of courses taught by the participants in the survey. [file 12302_2021_552_MOESM2_ESM.pdf]

Appendix S2. List of courses taught by the participants in the survey

**Agrarian Sciences**	Agricultural Sciences and Climate Change<br>Climate Change and Agricultural Productivity<br>Climate Change and Agriculture<br>Climate change, Gender and Sustainable Development<br>Economics of climate change
**Biological Sciences**	Water quality: management of a natural resource
**Business studies**	Business Sustainability<br>Human Values and Ethics<br>Corporate Sustainability Management<br>Sustainability Accounting
**Engineering**	Agricultural Meteorology<br>Air, Energy, Noise and Climate Change in Sustainable Cities<br>Climate Change and Disaster Risk Management<br>Dryland Management<br>Environmental and Energy Management<br>Green House Effect vs Infrared Radiation of the Artificial Surfaces<br>Integrated Water Resources Management<br>Sustainable Manufacturing
**Environmental and Earth Sciences**	Climate change adaptation, vulnerability and impacts<br>Civil Protection and Risk Management<br>Climate Change<br>Climate Change Adaptation and Project Design<br>Climate change and Sustainable Development<br>climate change is been tough in several courses such as Sustainable Development, Waste Strategies, Renewable energy, climate change etc<br>Climate Change Modelling<br>Climate Change, Adaptation and Development<br>Climate Change, Climatology<br>Climate change, Vulnerability and Adaptation<br>Climate Change: Impacts, Adaptations and Mitigations, General Climatology, Agricultural Meteorology, Atmospheric General Circulation, Atmospheric Dynamics, The Science of Climate Change.<br>Climate Resilient Development<br>Environmental and Social Sustainability<br>Environmental Economics and Policy<br>Environmental Education<br>Environmental Policies and Regulations<br>Geomorphology, Landscape Ecology<br>Global Climate Crisis, Global Climate Change, Physical Geography, Landscape Analysis and Green Infrastructure<br>Green economy<br>Introduction to climate change; Climate change and global health; Climate change and law<br>Land use and climate change; Climate change challenges and responses<br>Masters degree in Climate change and sustainable development<br>MSc in Carbon Management<br>Population dynamics and environmental change<br>Research methods<br>Several courses. few lectures in Year 1 (on climate change as a global environmental issue), For third year students, there are 2 courses, namely, 1. Climate Change, and 2. Adapting business for Climate Change. We have a postgraduate (MSc/PG Diploma) program on Climate Change and Environmental Management with 12 modules/courses within that program. All the above courses/programs are the ones taught in the Faculty of Science. Climate change-related courses are also taught in the Faculties of Arts, Technology, etc., as well.<br>Technology, Society and Globalisation: Sustainability, the 21st century challenge
**Humanities/Linguistics**	Imagining Futures in the Anthropocene
**Social Sciences**	"Society-Economy-Globalization", "Political Eco-Geography", a short course on Conflict Minerals & Bilateral Investment Treaties<br>Climate Adaptation; Climate Fiction; Climate Communication; Environmental Justice in Disasters<br>Climate change adaptation in spatial planning<br>Climate, Energy and society<br>Science and Risk Communication<br>Community-Based Disaster Risk Reduction and Management

	Contemporary challenges in international relations
	Corporate Social Responsibility
	Disaster Risk Reduction in Cities
	Eco-innovation
	Economics of the Global South
	EdD learning pedagogy and diversity
	Environment & Society and "Ecological Economics"
	Environment and Society. Social Science and Social Problems
	Environmental and Resource Economics
	Environmental Inequalities/Environmental Justice
	environmental politics
	History of Climate Change and the impact on humam societies
	MSC Climate Change and Environmental Policy
	MSc in Climate Change: Policy, Media and Society
	Only as examples in courses on Environmental Education and Environmental Interpretation, not direct topic of either course.
	Planning for disaster preparedness and management
	Planning for Environmental Change
	Climate Change, Population Vulnerability and Adaptation
	Population and Environment, EIA, Environmental Economis, Natural Resource Economics
	Social Movements (two courses)
	Society in the Arctic
	Sustainable Development
	Norway's energy transitions: Policy directions and challenges
	The Climate Emergency
	Transportation GEOGRAPHY
**Others**	Agro-Climatology
	Architecture Studio C (Environmental Design)
	Business Sustainability
	Curriculum and Pedagogy: HASS; Introduction to History
	economics of climate change
	Environmental, Health and Climate Change
	Forestry and Climate Change
	Introduction to Environmental Studies (for non-majors); Intro to Env Studies (for majors); ENV 208: Climate Change: Causes, Consequences and Solutions; ENV 398: Climate Change and Conservation; ENV 328: Environmental Pollution; ENV318 Biodiversity Conservation
	Meteorology and climatology, Applied climatology, Soil Health & Climate Change, Climate change, adaptation and mitigation, Agrometeorology and response farming, Agrometeorological Instruments and methods of observation
	Methods for energy system & energy policy assessment, cities in transition etc.
	Paradigms of Development & Environmental Educaton and Communication
	Planning for Climate Change
	Rural Sociology 450 - Environmental Sociology
	Sustainable development and business
	We have dedicated societies that work for climate change
